# Peptide ES15-1 derived from *Haemonchus contortus* promotes goat Th17 response by regulating the STAT3/RORγt pathway

**DOI:** 10.1186/s13567-025-01649-y

**Published:** 2025-11-19

**Authors:** Cheng Chen, Jiajun Feng, Jilata Amu, Zhaohai Wen, Yangchun Tan, Yongde Xu, Xianglin Pu, Mingmin Lu, Xiaokai Song, Lixin Xu, Xiangrui Li, Ruofeng Yan

**Affiliations:** 1 Ministry of Education (MOE) Joint International Research Laboratory of Animal Health and Food Safety, College of Veterinary Medicine, Nanjing Agricultural University, Nanjing, 210095 People’s Republic of China; 2 Department of Veterinary Medicine, College of Coastal Agricultural Sciences, Guangdong Ocean University, Zhanjiang, 524088 People’s Republic of China

**Keywords:** *Haemonchus contortus*, ES-15, Th17 response, STAT3/RORγt pathway

## Abstract

Th17 cells play important roles in anti-infective responses. The 15 kDa excretory/secretory protein of *Haemonchus contortus* (HcES-15) has been identified as a promising immune-protective antigen against *H. contortus* infection capable of up-regulating IL-17, IL-4 and IL-10 production. To obtain the peptides that primarily induce the Th17 immune response, we amplified and expressed the peptides ES15-1, ES15-2 and ES15-3 from HcES-15. In vitro studies demonstrated that ES15-1 stimulated transcriptional activation of the STAT3/RORγt signaling pathway and induced IL-17 production in goat peripheral blood mononuclear cells (PBMCs). In vivo studies, flow cytometric analysis revealed that subcutaneous injection of PLGA-encapsulated ES15-1 peptide (PLGA-ES15-1, 50 μg) significantly enhanced Th17 cell differentiation in the spleens of BALB/c mouse. Consistent with these findings, ELISA quantification demonstrated that ES15-1 treatment significantly increased serum levels of pro-inflammatory cytokine (IL-17, IL-1, IL-6, and TNF-α). In goat immune protection studies, goats (*n* = 6) were subcutaneously immunized with 500 μg of PLGA-ES15-1 on days 0 and 14, followed by infection with *H. contortus* infective third-stage larvae (iL3s) 1 week post-second immunization. ES15-1 significantly enhanced serum levels of pro-inflammatory cytokines (IL-17, IL-1, IL-6, TNF-α). At autopsy, vaccinated goats exhibited 69.0% (*p* < 0.001) reduction of fecal egg counts (FEC) and 50.54% (*p* < 0.05) reduction of worm burdens versus controls. Our findings suggested that peptide ES15-1 enhanced Th17 responses through regulation of the STAT3/RORγt pathway, conferring a certain immune protection against *H. contortus* infection.

## Introduction

*Haemonchus contortus* is widely recognized as one of the most pathogenic nematodes in grazing-based production systems. Haemonchosis, caused by *H. contortus*, results in substantial economic losses estimated at tens of billions of dollars annually [1, 2]. *H. contortus* primarily parasitizes the abomasum of sheep and goats, causing gastroenteritis and digestive dysfunction*.* It can lead to emaciation, wasting, anemia, and death in severe cases [1, 3, 4]. This nematode’s pathogenicity stems from its blood-feeding behavior and the immunomodulatory excretory-secretory proteins (ESPs) [5–7].

Antigens of *H. contortus* can be classified into two categories based on immunogenic properties: native antigens and hidden antigens. Native antigens, naturally recognized by the host immune system during infection, induce specific humoral and cellular responses, including excretory-secretory proteins (e.g., HcESPs), cuticular antigens (e.g., Hc-sL3), and gut-specific antigens. Among the ESPs of *H. contortus*, HcES-15 has been identified as a key component with significant antigenic properties [7]. Vaccination of sheep with a combination of HcES-15 and the 24 kDa excretory-secretory protein of *H. contortus* (HcES-24) resulted in 72.9% and 82.2% reductions in mean FEC and worm burdens, respectively [8]. The recombinant HcES-15 (rHcES-15) and HcES-24 (rHcES-24) reduced FEC by 49% and worm burdens by 55% in 9-month-old sheep. However, rHcES-15 and rHcES-24 did not offer protection in 3-month-old lambs [9]. Previous studies from our laboratory demonstrated that incubation of rHcES-15 with PBMCs stimulated the production of cytokines (IL-17, IL-4, and IL-10) [10]. Given the potential of HcES-15 to induce Th17, Th2, and Treg immune responses, we expressed this protein in fragments to identify peptides predominantly eliciting Th17 immunity for evaluating its role against *H. contortus* infection.

Th17 cells enhance host defense against extracellular pathogens through secretion of effector cytokines (IL-17, IL-21, and IL-22), which mediate potent recruitment and activation of neutrophils. Although the precise mechanisms of Th17 immune responses in combating *H. contortus* infection remain incompletely understood, multiple studies have demonstrated their critical immunomodulatory role in host defense against this parasite. Esmaeil et al. were the first to identify 18 single nucleotide polymorphisms (SNPs) associated with *H. contortus* resistance in Florida Native sheep, including key Th17 pathway genes STAT3 and IL2RB (IL-2 receptor β chain) [11]. Analysis of protein–protein interactions (PPIs) between caprine innate immune signaling proteins and *H. contortus* excretory-secretory proteins (ESPs) revealed that the NOD-like receptor (NLR) and IL-17 signaling pathways exhibited the highest number of molecular interactions [12]. Emerging evidence demonstrates that infection with *H. ontortus* L3 larvae significantly enhances IL-17 secretion in host animals [13]. Multiple studies have further confirmed that recombinant forms of *H. contortus* excretory-secretory proteins (ESPs) and specific parasite antigens can markedly upregulate IL-17 transcription levels in caprine PBMCs [14, 15].

Th17 cells, a subset of CD4^+^ T cells, are characterized by their high secretion of interleukin-17 (IL-17) following activation [16]. IL-17 is an early promoter of T-cell-induced inflammatory responses that amplifies the inflammatory response by promoting inflammatory factors. The IL-17 cytokines family, consists of six member ligands (IL-17A~IL-17F) and five receptors (IL-17RA~IL-17RD and SEF). After binding to the receptor, IL-17 exerts its biological effects through NF-κB translocation and MAPK phosphorylation(p-ERK/p-JNK/p-p38) [17]. The trimer which bound with the dimer of NF-κB and IκB is activated when stress and inflammatory stimuli induce phosphorylation of IκBα by the IKK complex, leading to dissociation of the NF-κB dimer. Activated IKK phosphorylates the n-terminal serine of IκB and allowing NF-κB translocated into the nucleus to regulate gene expression as a transcription factor [7, 18, 19]. Subsequently, Th17 cells secrete IL-17A, IL-17F, IL-6, and TNF-α, which collectively mobilize, recruit, and activate neutrophils. IL-17 can effectively mediate the excitation process of neutrophil mobilization, thus effectively mediating the inflammatory response of tissues [20, 21].

## Materials and methods

### Parasites and animals

The *Haemonchus contortus* strain used in this study was originally isolated from Inner Mongolia, China, and maintained through serial passage in goats. Feces (~1 kg) from *H. contortus* infected-goat were collected, crushed, mixed with water, and combined with vermiculite to make the mixture moisture. The mixture was placed in a glass dish (16 cm diameter), which was covered with aluminum foil perforated with several holes to allow air flow and incubated at 28 °C for 7 days. The larvae were recovered by filtering the mixture through cheesecloth, identified microscopically, and preserved at 4 °C in penicillin (100 IU/mL) treated water until use [22].

Eighteen healthy 6-month-old Boer × Local goat crossbreds were obtained from the original breeding farm in Lai’an County, Anhui Province, China. The animals were housed in the College of Veterinary Medicine’s experimental facility under controlled conditions, provided with sterilized hay, corn-based feed, and ad libitum access to water.

Sixty female BALB/c mice (4–6 weeks old) were obtained from Qinglongshan Animal Breeding Farm (Jiangning District, Nanjing) and housed in animal center. All animals received standardized care in compliance with institutional ethical guidelines (Official Notice on Standardizing the Management and Care of Laboratory Animals Nanjing Agricultural University, 2018).

### Molecular cloning and expression of peptide ES15-1, ES15-2 and ES15-3

The complete open reading frame(ORF) of HcES-15 (GenBank accession No.: AY821552.1) was retrieved from the NCBI database. T-cell epitopes and antigenicity index of HcES-15 were predicted using DNASTAR Protean software. Based on the epitope analysis, three peptide fragments (ES15-1, ES15-2, and ES15-3) were designed, and their corresponding genes were amplified by PCR using primers listed in Table 1. The forward and reverse primers contained the initiation codon (ATG) with *Bam*H I restriction site at the 5’-end and the termination codon (TAA) with *Hin*d III restriction site at the 3’-end. The preserved recombinant plasmid rHcES-15 [10, 23] was used as the template for polymerase chain reaction (PCR) amplification of each peptide. The PCR reaction mixture consisted of 1.0 μL template DNA, 25 μL PrimeSTAR Max Premix (2X), 2 μL forward primer (10 pM), 2 μL reverse primer (10 pM), and nuclease-free water added to 50 μL. The reaction conditions were as follow: predenaturation at 94 °C for 1 min, denaturation at 98 °C for 10 s, annealing at 55 °C for 5 s, extension at 72 °C for 5 s, for total of 35 cycles. PCR products were purified using the E.Z.N.A.^®^ Gel Extraction Kit (Omega Bio-Tek, USA) and cloned into the *Bam*H I / *Hin*d III sites of the pET-32a expression vector (TaKaRa Biotechnology, China). The ligated constructs were transformed into *Escherchia. coli* BL21(DE3) competent cells. Positive clones were verified by dual restriction enzyme digestion (*Bam*H I / *Hin*d) and confirmed by Sanger sequencing (Tsingke Biological Technology, Nanjing, China). Recombinant protein expression was induced in LB medium (containing 100 μg/mL ampicillin) using 1 mM isopropyl β-D-1-thiogalactopyranoside (IPTG) at 37 °C for 5 h. Bacterial cells were lysed, and His-tagged fusion proteins were purified from the supernatant using a His-Bind® Resin Chromatography Kit (Novagen, USA). The purified proteins were dialyzed against PBS (pH = 7.4) to remove residual imidazole, followed by endotoxin removal using the ToxinEraser™ Endotoxin Removal Kit (GenScript, USA). Protein purity and concentration were assessed by 10% SDS-PAGE with Coomassie Brilliant Blue R-250 staining.

**Table 1:** **Primers used for plasmid construction**

Gene name	Primer sequence (5′–3′)	Restriction
ES15-1-f	CG**GGATCC**ATGTTCTTCGCTTTTGCAGT	*Bam*H I
ES15-1-r	CCC**AAGCTT**TTACTTAGCCGCCAAGTCAT	*Hin*d III
ES15-2-f	CG**GGATCC**ATGGCAATGGTGGAAGC	*Bam*H I
ES15-2-r	CCC**AAGCTT**TTAGTTCAGCAGAACCAAGC	*Hin*d III
ES15-3-f	CG**GGATCC**ATGCCCTTCAAAAAATATGC	*Bam*H I
ES15-3-r	CCC**AAGCTT**TTAGTTGGGGGTATTGTAGAC	*Hin*d III

f: Forward primer sequence, r: Reverse primer sequence. The restriction enzyme cutting sites are highlighted in bold.

### Effects of peptide ES15-1, ES15-2 and ES15-3 on Th17 response in goat PBMCs

Freshly isolated PBMCs (5 × 10⁶ cells/mL) from healthy goats using human lymphocyte separation solution [23] were resuspended in complete RPMI 1640 medium containing 100 U/mL penicillin, 100 μg/mL streptomycin, and 10% FBS. Cells were seeded in 12-well plates (2 mL/well) and stimulated with 40 μg/mL of peptides (ES15-1, ES15-2, ES15-3) for 24 h at 37 °C in a 5% CO₂ humidified incubator [10, 24, 25]. Control groups included cells treated with an equal volume of pET-32a carrier protein and sterile PBS or left unstimulated (Blank). Post-incubation, total RNA (1 μg) was extracted using RNA isolator (Total RNA Extraction Reagent) and quantified via Nanodrop One spectrophotometry (Thermo Fisher Scientific, Model ND-ONE-W, USA). cDNA synthesis was performed using the Evo M-MLV Mix Kit with gDNA Clean for qPCR (Accurate Biology, AG11728, China) according to the manufacturer’s protocol.

mRNA levels associated with Th immune subsets were quantified by qPCR. Target genes including IL-17 (Th17), IFN-γ (Th1), IL-4/IL-5/IL-13 (Th2), IL-9 (Th9), IL-21 (Tfh), IL-22 (Th22), TGF-β/IL-10 (Treg). The qPCR reaction mixture (10 μL total volume) is as follows: Template 1.0 μL, SYBRGreen ProTaq HS premixed 5 μL, forward primer (10 pM) 0.2 μL, reverse primer (10 pM) 0.2 μL, and added nuclease-free ddH_2_O to adjust the final volume. The qPCR cyclic condition was as followed: initial denaturation at 95 °C for 30 s, denaturation at 95 °C for 10 s, annealing/extension at 60 °C for 30 s, 35 cycles. Relative transcript levels were calculated using the 2^−ΔΔCt^ method, with β-actin (amplification efficiency E = 95.94%, R^2^ = 0.9964) as reference genes (primers details in Table 2) [26].

**Table 2:** **Primers used for qPCR**

Gene name	Forward primer sequence (5′–3′)	Reverse primer sequence (5′–3′)
IL-17	TTGTAAAGGCAGGGGTCATC	GGTGGAGCGCTTGTGATAAT
IFN-γ	GAACGGCAGCTCTGAGAAAC	GGTTAGATTTTGGCGACAGG
IL-4	GTACCAGCCACTTCGTCCAT	GCTGCTGAGATTCCTGTCAA
IL-5	GCTGCCTATGTTTGTGCCAATGC	GATGCGTGGAGAGCAGTGTCAAG
IL-13	CCTGTGTTGTTCTAGGCTCCA	CTCCACACCATGCTGCCATT
IL-9-	GCACGAGATTCCCCCTGATT	CGTGTTGCCTGTTGTGGTTT
IL-21	AGCTCCAGAAGATGTAAAGAGACA	CATTTGTGGGAGGCAGTTTCC
IL-22	TCCAGGGAATCAATCAGGTGAC	CGTTTCTCTGGATGTGCTCG
TGF-β	GAACTGCTGTGTTCGTCAGC	TCCAGGCTCCAGATGTAAGG
IL-10	CCTTGTCGGAAATGATCCAG	AGGGCAGAAAACGATGACAG
IL-6	CGTCGACAAAATCTCTGCAA	TTCCCTCAAACTCGTTCTGG
IRF4	AAGTGTACCGGATCGTCCCC	GGGATCATGTAGTTGTGAACCTGCT
STAT3	CGAAGCTGACCCAGGTAGTG	TATTGCTGCAGGTCGTTGGT
BATF	AGATCAAGCAGCTCACGGAA	CGTGAGTCTGGTGGAAGGCA
RORγt	GCCAGGGCTCCAAGAGAAAA	CGATAGGGTGGAGATGCTGG
IL-23	GTCCCCCGTATCCAGTGTGA	CACAGGGCCATCTGGGAATA
MyD88	AAGTTTGCACTCAGCCTCTC	ACAGTGATGAAGCGCAGGATG
NF-κB	GAGATGCCACTGCCAACAGATG	CTTCACACACATAACGGAAACGA
IL-1	GTGCTGGATAGCCCATGTGT	CAGAACACCACTTCTCGGCT
STAT1	ATGCCACCGAACTTACCCAG	AGCTGATCCAGGCAAGCATT
GATA3	ATGCAAGTCGAGGCCCAAG	GGTAATGCCCGGTTCCATCT
Foxp3	ACCTGGAAGAATGCCATCCG	AACTCATCCACGGTCCACAC
β-actin	GGACTTCGAGCAGGAGATGG	AAGGAAGGCTGGAAGAGAGC

### Effects of peptide ES15-1 on STAT3/RORγt pathway

PBMCs (5 × 10^6^/mL) from healthy goats were incubated with 20 μg/mL peptide ES15-1 in a carbon dioxide cell incubator at 5% CO_2_, 37 °C, for 6 h. Equal volume of sterile PBS was added to unstimulated (Blank) was used as controls. Total RNA of PBMCs was extracted by RNA isolator (Total RNA Extraction Reagent) and reverse-transcribed to obtain cDNA using Evo M-MLV Mix Kit with gDNA Clean for qPCR (Accurate Biology, AG11728, China). Transcript levels of Th17-associated markers were quantified using SYBR Green-based qPCR. Targets genes included STAT3 pathway (IL-6, IRF4 and STAT3) and RORγt pathway (TGF-β, BATF and RORγt), and the Th17 cell differentiation cytokine IL-23. Primers for all targets genes (Table 2) were designed with Primer Premier 5.0 (Premier Biosoft, USA). Relative gene expression was normalized to housekeeping gene (β-actin) and calculated using the 2^−ΔΔCt^ method. Meanwhile, culture supernatants were assayed for IL-17 levels using IL-17/IL-17A ELISA Development kit (Novus, USA).

To further investigate the immunomodulatory effect of ES15-1, the transcriptional changes in the following immune-related pathways were assessed: NF-κB and MAPK pathway (MyD88, NF-κB and IL-1), Th1 (STAT1), Th2 (GATA3) and Treg (Foxp3). Primers for all targets (Table 2) were designed with Primer Premier 5.0. qPCR conditions and data analysis followed the same methodology as above.

### Preparation of nanoparticles (NPs) PLGA-ES15-1

Fifty milligrams of poly (lactic-co-glycolic acid) (PLGA) were dissolved in 1 mL dichloromethane (DCM), followed by addition of 2 mL 5% (w/v) polyvinyl alcohol (PVA) to form a water-in-oil (w/o) emulsion. Utilizing an ultrasonic processor (JY92-IIN, NingBo Scientz Biotechnology, Ningbo, China) for a 5 min duration (40 W, 5 s on, 5 s off), while maintaining an ice bath. During this process, 5 mg of ES15-1 peptide or pET-32a carrier protein (control) was added dropwise under continuous agitation. This w/o emulsion was subsequently introduced into the external aqueous phase, comprised of a 5% PVA solution in deionized water. The same sonication conditions were applied to obtain the final emulsion, forming a water–oil-water (w/o/w) configuration. The organic solvent within the emulsion was eliminated through evaporation under magnetic stirring for a duration of 4–5 h in a chemical fume cupboard, conducted at room temperature. The resultant antigen-loaded nanoparticles (PLGA-ES15-1 and PLGA-pET-32a) were separated from the nanoparticle (NP) solution through centrifugation at 30 000 *g* for 30 min at 4 °C. The supernatant was collected to determine protein loading efficiency using the BCA Protein Assay Kit. At the same time, the precipitated NPs were subjected to two wash cycles via centrifugation with ultrapure water. The NPs were subsequently placed within a freeze-drying machine (Labconco™, Thermo Fisher Scientific, Waltham, MA, USA) for a period of 24 h and stored at −80 °C until they were ready for use in further experiments. To determine the encapsulation efficiency (EE) and loading capacity (LC) using the following equations:

$${\text{EE}}\left( \% \right){ }\,\, = \,\,{ }\frac{{{\text{Total protein }} - {\text{unbound protein}}}}{{\text{Total protein }}} \times 100\%$$

$${\text{LC}}\left( \% \right){ }\, = \,\,{ }\frac{{\text{Loaded protein}}}{{\text{Total mass of the nanovaccine}}} \times 100\%$$

A small amount of PLGA-ES15-1 and PLGA-pET-32a nanoparticles were extracted and analyzed for particle morphology using scanning electron microscope (SEM) Regulus 8100 (Hitachi, Tokyo, Japan) [27, 28].

### Effect of ES15-1 on Th17 immune response in mice

Total of 60 BALB/c mice were divided into two groups. Mice in each group were injected subcutaneously with 50 μg PLGA-ES15-1 and PLGA-pET-32a, respectively.

Th17 response related cytokines were detected by ELISA. Five mice in each group were randomly selected to collect whole blood from the ocular globe and isolate serum once a week. The secretion of cytokines IL-12, IFN-γ, IL-4, TGF-β, IL-17, IL-6, IL-1 and TNF-α in the serum of mice at 1w-6w were detected by ELISA kit acquired from Nanjing Lapuda Biotechnology Co., Ltd (Nanjing, China).

Th17 cells of mice were detected by flow cytometry. The spleen of mice was extracted under aseptic conditions and cut up with scissors. Single cell suspension was obtained by crushing the spleen in a 70 μm cell filter. Mouse PBMCs were collected according to the instructions of mouse splenic lymphocyte isolation kit. The concentration of PBMCs collected from mice was adjusted to 10^6^ /mL and added into the 6 well cell culture plate. Each well was added 2 μL of 500 × Cell Stimulation Cocktail containing protein transport inhibitors and culture in a 5% CO_2_ cell incubator at 37 °C for 4–6 h. Cells were collected in a 1.5 mL Eppendorf (EP) tube, centrifuged at 4 °C, 500 *g*/min for 10 min. Wash and re-suspend PBMCs with aseptic PBS and centrifuge at 4 °C, 500 *g*/min for 10 min to discard the supernatant and repeat twice. For cell surface staining, cells were incubated with CD3e Monoclonal Antibody and CD4 Monoclonal Antibody at 4 °C for 30 min in the dark. Cells were wash and re-suspend PBMCs with sterile PBS and repeated twice. 100 μL sterile PBS was used to suspend the cells and 100 μL Intracellular Fixation Buffer added to fix the cells and keep in the dark at room temperature for 20–60 min. Wash the cells twice by using 2 mL 1 × Permeabilization Buffer. The cells were re-suspended with 100 μL 1 × Permeabilization Buffer and incubated with IL-17A Monoclonal Antibody in the dark at room temperature for 20–60 min. Then wash the cells twice by using 2 mL 1 × Permeabilization Buffer. The cells were re-suspended with 100 μL Flow Cytometry Staining Buffer, the cells were examined by CytoFLEX (Beckman Coulter, USA) and the data was analyzed with the CytoExpert (Default-Configuration).

### Effect of ES15-1 on IL-17 immune response in goat

Twelve goats were divided into two groups, and injected subcutaneously with 500 μg PLGA-ES15-1 and PLGA-pET-32a respectively. The second immunization was given 2 weeks after the first immunization. Followed by each goat was orally infected with 6000 *H. contortus* iL3s 1 week after the second immunization.

Th17 response related cytokines were detected by ELISA. Serum samples were collected from goats via jugular vein weekly (pre-immunization serum as 0 w). The concentration of cytokines of IL-12, IFN-γ, IL-4, TGF-β, IL-6, IL-1 and TNF-α at 0 week (w), 1 w, 2 w and 3 w were detected according to the manufacturer of ELISA kit acquired from Nanjing Lapuda Biotechnology Co., Ltd (Nanjing, China). The concentration of cytokine IL-17 in serum of goats were detected by IL-17/IL-17A ELISA Development kit (Novus, USA).

### Reductions in fecal egg shedding and worm burdens

Fecal egg count expressed as eggs per gram (EPG), were monitored every two days starting 14 days post-infection (dpi) using the McMaster method.

The number of female and male adult worms in abomasum were measured following necropsy on the day 56 of the trial.

### Statistical analysis

All data were analyzed and visualized using GraphPad Prism 6.0 (GraphPad Software, USA). The results are presented as Mean ± Standard Error of the Mean (SEM). Differences among different groups were analyzed by one-way analysis of variance (ANOVA), and significance was expressed as *P < 0.05, **P < 0.001, ***P < 0.0001, ****P < 0.00001.

## Results

### Molecular cloning and expression of peptide ES15-1, ES15-2 and ES15-3

**Fig. 1:**
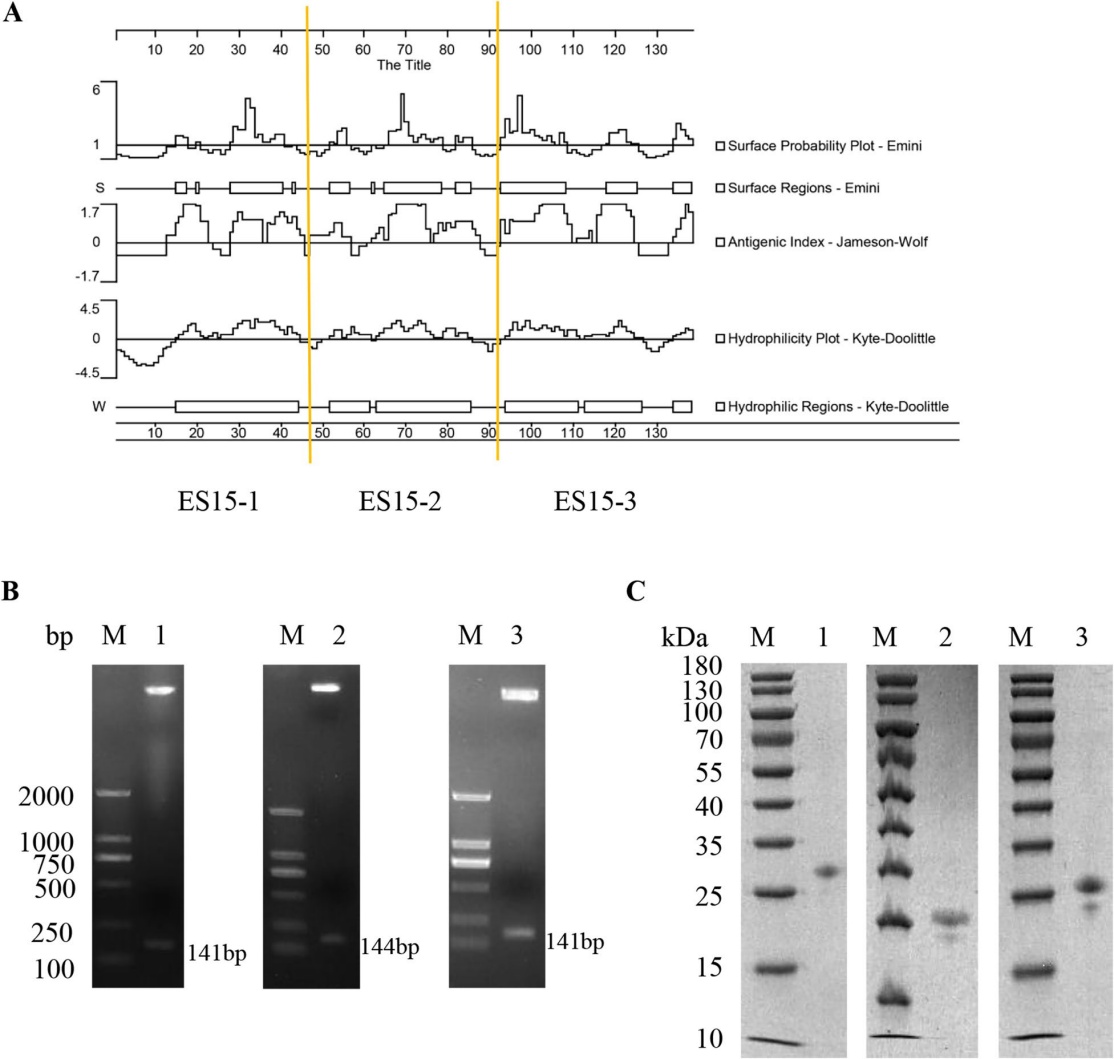
**Molecular cloning and expression of peptide ES15-1, ES15-2 and ES15-3.** **A** Evaluation of the T cell epitope and antigen index of HcES-15 by DNASTAR (Protean) software. (a) Peptide ES15-1 (Amino acids 1~46 of HcES-15). (b) Peptide ES15-2 (Amino acids 47~93 of HcES-15). (c) Peptide ES15-3 (Amino acids 94~138 of HcES-15). **B** Gene encoding peptide ES15-1, ES15-2 and ES15-3 was cloned into pET-32a and confirmed by restriction enzyme digestion. M: DNA Marker DL2000 1: pET32a-ES15-1 2: pET32a-ES15-2 3: pET32a-ES15-3. **C** Purified peptides were analyzed by SDS-PAGE. M: Standard protein molecular weight marker 1: pET-32a ( +)-ES15-1 2: pET-32a ( +)-ES15-2 3: pET-32a ( +)-ES15-3.

According to the analysis of T-cell epitope prediction (Surface Regions and Antigenic Index) and hydrophilicity (Hydrophilic Regions), HcES15 was divided into three immunogenic peptides (ES15-1, ES15-2, and ES15-3) according to Surface Regions-Emini (Figure 1A). Genes encoding peptide ES15-1, ES15-2 and ES15-3 were amplified by PCR from the HcES-15 recombinant plasmid, yielding 141 bp, 144 bp and 141 bp fragments. These fragments were cloned into the prokaryotic expression vector pET-32a ( +), confirmed by restriction enzyme digestion and agarose gel electrophoresis (Figure 1B). The peptide ES15-1, ES15-2, and ES15-3 expressed in *E. coli* BL21(DE3) as a His 6 tagged fusion protein were purified via affinity chromatography. The expressed ES15-1, ES15-2 and ES15-3 product were about 25 kDa and confirmed by SDS-PAGE after staining with Coomassie brilliant blue (Figure 1C).

### ES15-1, ES15-2 and ES15-3 induce variant Th immune responses in goat PBMCs

**Fig. 2:**
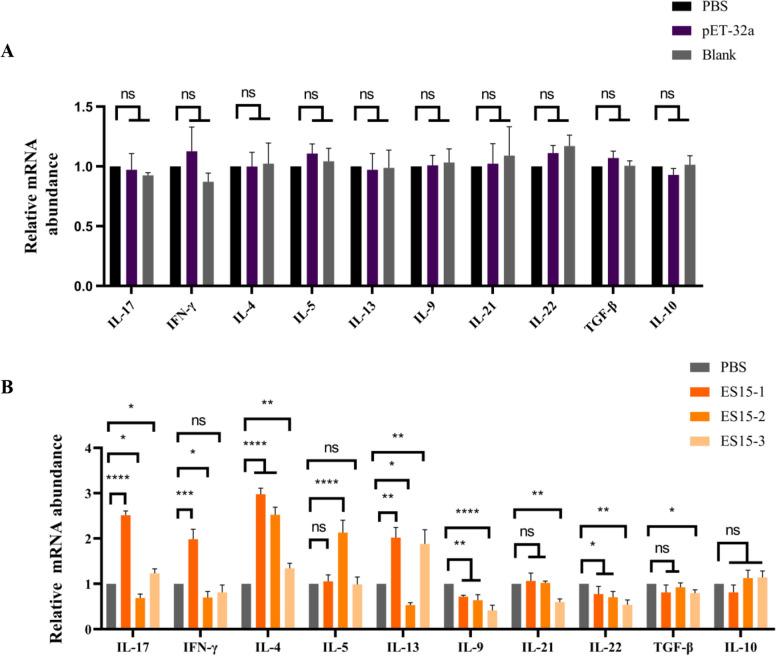
**Effect of peptide ES15-1, ES15-2 and ES15-3 on Th17 and other T cell subtype responses in goat PBMCs**.** A** Relative mRNA abundance of IL-17, IFN-γ, IL-4, IL-5, IL-13, IL-9, IL-21, IL-22, TGF-β and IL-10 were detected by qPCR. Goat PBMCs were incubated with carrier protein (pET-32a), PBS (PBS) or water (Blank). **B** Effects of peptide ES15-1, ES15-2 and ES15-3 on the transcription of IL-17, IFN-γ, IL-4, IL-5, IL-13, IL-9, IL-21, IL-22, TGF-β and IL-10. Goat PBMCs were incubated with ES15-1, ES15-2, ES15-3 or PBS. The relative mRNA abundance was detected by qPCR. Data are presented as Mean ± SEM. *P*-values < 0.05 (*), < 0.01 (**), < 0.001 (***), and < 0.0001 (****) were considered statistically significant. ns represented no significance.

No significant differences were found in cytokine transcription levels between the pET-32a carrier protein and PBS control groups (Figure 2A). In the group of ES15-1 co-incubated with PBMCs, the expressions of IL-17, IFN-γ, IL-4 and IL-13 were significantly up-regulated, IL-9 and IL-22 were significantly down-regulated, while IL-5, IL-21, TGF-β and IL-10 showed no significant difference when compared with control group (PBS). For ES15-2 group, the expressions of IL-4 and IL-5 were significantly up-regulated and IL-17, IFN-γ, IL-13, IL-9 and IL-22 were significantly down-regulated. IL-21, TGF-β and IL-10 showed no significant difference compared with control group. For ES15-3 group, the expressions of IL-17, IL-4 and IL-13 were significantly up-regulated and IL-9, IL-21, IL-22 and TGF-β were significantly down-regulated. While IFN-γ, IL-5 and IL-10 showed no significant difference compared with control group (Figure 2B). These results indicated that peptide ES15-1 emerged as the most potent inducer of Th17 immunity, with IL-17 transcription significantly elevated compared to other peptides. ES15-2 exhibited immunosuppressive effects on multiple pro-inflammatory pathways (Th17/Th1/Th9/Th22). ES15-3 showed a mixed immunomodulatory profile, promoting Th17/Th2 activity while suppressing Th9/Tfh responses. Among the three peptides, peptide ES15-1 mainly upregulates IL-17 transcription.

### ES15-1 induce goat Th17 response through STAT3/RORγt pathway

**Fig. 3:**
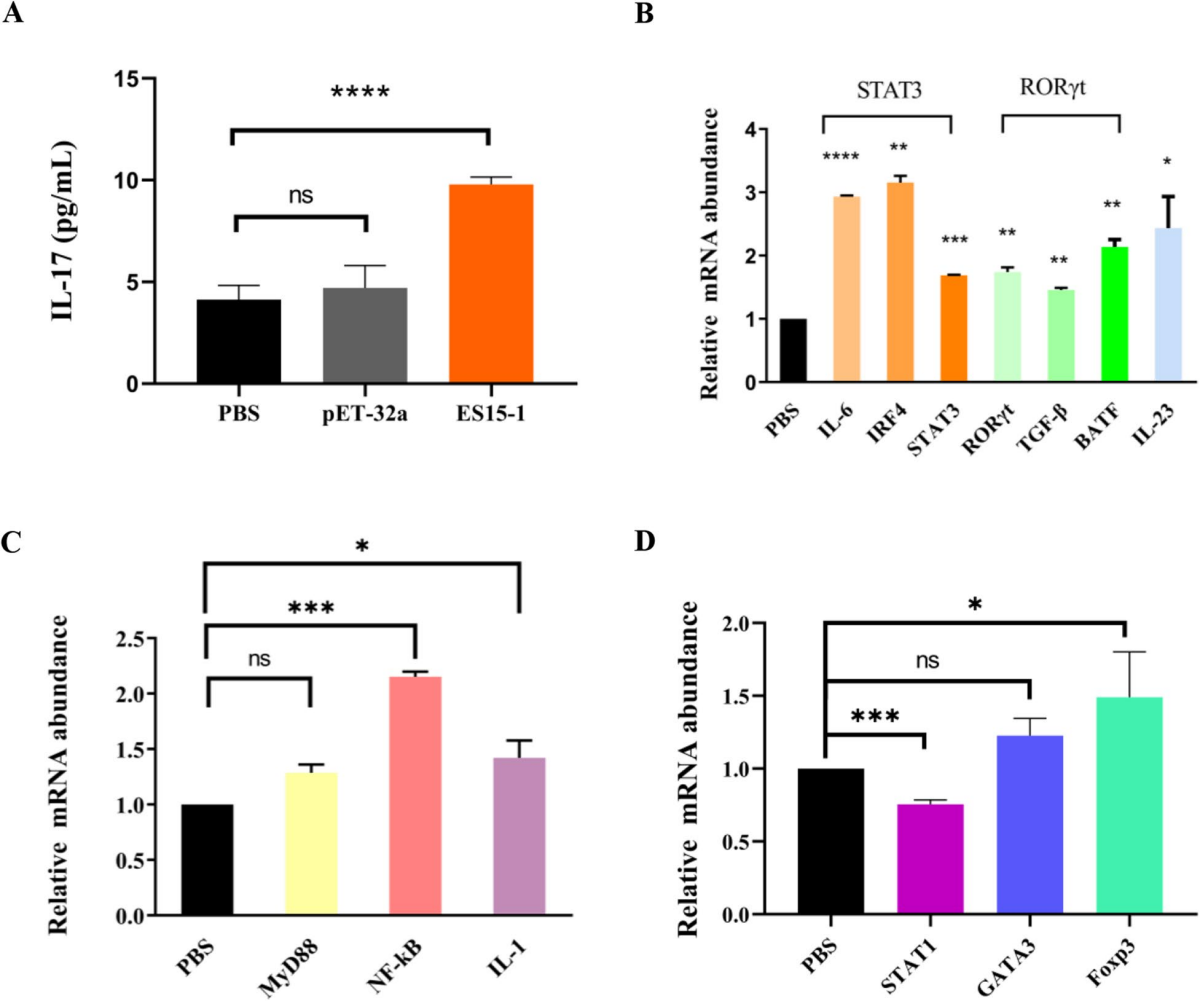
**Peptide ES15-1 induce Th17 response through STAT3/RORγt pathway in goat PBMCs.** **A** ES15-1 promoted the secretion of IL-17. **B** ES15-1 promoted the transcription of cytokines associated with Th17 cell differentiation pathway. **C** ES15-1 promoted the transcription of and IL-1through NF-κB/MAPK pathway. MyD88: adaptor protein NF-κB: Transcription Factor IL-1: Inflammatory Cytokine **D** ES15-1 induced the key transcription factors Foxp3 of Treg cell in PBMCs. Data are presented as Mean ± SEM. *P*-values < 0.05 (*), < 0.01 (**), < 0.001 (***), and < 0.0001 (****) were considered statistically significant. ns represented no significance.

Peptide ES15-1 significantly increased IL-17 secretion in goat PBMC when compared to controls (Figure 3A). Transcription levels of Th17 associated markers (IL-6, IRF4, STAT3, TGF-β, BATF, RORγt, and IL-23) were markedly upregulated in ES15-1-treated PBMCs (Figure 3B). It indicated that ES15-1 promotes Th17 cell differentiation by upregulating key transcriptional regulators (RORγt and STAT3) and driving IL-17 production.

The transcription levels of MyD88, NF-κB and IL-1 associated with NF-κB and MAPK pathway were also detected to further validate the inflammatory responses. The result showed that the expression of NF-κB and IL-1 were significantly up-regulated while MyD88 was not significantly different from the control group (Figure 3C). This suggests that ES15-1 induces inflammatory responses in PBMCs through NF-κB pathway activation, independent of MyD88 signaling, leading to IL-1β secretion.

Additionally, the key transcription factors in Th1, Th2 and Treg cell differentiation pathways were also examined. The results showed that STAT1 was significantly down-regulated, Foxp3 was significantly up-regulated, and GATA3 was not significantly different from the control group (Figure 3D). These findings suggest that, Th1 cells differentiation was inhibited while Treg cells differentiation was promoted in PBMCs incubated with peptide ES15-1.

### ES15-1 promoted Th17 response in mice

**Fig. 4:**
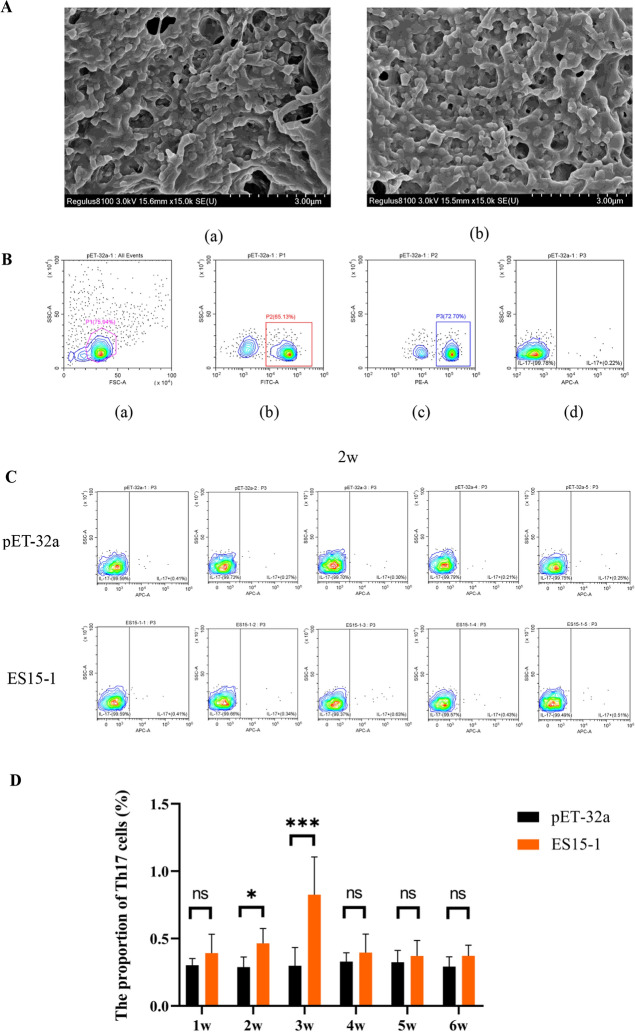
**ES15-1 upregulated Th17 cell differentiation in mice.** **A** ES15-1 was coated with PLGA nanoparticle. (a) PLGA-pET-32a (b) PLGA-pET-32a-ES15-1 **B** Illustration of Th17 cell analysis using flow cytometry. (a) The graphic displays the percentage of live cells from all cells. 75.94% live cells were circled. (b) The graphic displays the percentage of CD3 positive cells for detection of T cells. 65.13% CD3 positive cells were identified. (c) The graphic displays the percentage of CD4 positive cells for detection of CD4 cells. 72.70% CD4 positive cells were identified. (d) The graphic displays the percentage of IL-17 positive cells for detection of Th17 cells. 0.22% IL-17 positive cells were identified. **C** Th17 cells differentiation in spleen was detected by flow cytometry at 2w. **D** Th17 cells ratio in spleen of mice treated with PLGA-pET-32a and PLGA-pET-32a-ES15-1. Data are presented as Mean ± SEM. *P*-values < 0.05 (*) and < 0.001 (***) were considered statistically significant. ns represented no significance.

SEM analysis confirmed the successful preparation of PLGA-pET-32a and PLGA-ES15-1 subunit vaccines. The particles exhibited smooth, spherical morphology with a uniform size distribution of 150–300 nm (Figure 4A).

Flow cytometry analysis revealed a progressive increase in the Th17 cell differentiation ratio within the spleens of mice during the first three weeks. Notably, Th17 cell proportions were significantly elevated compared to the control group at 2 weeks (2w) and 3 weeks (3w) (*P* < 0.05), while no significant differences were observed at 1 week (1 w) and 4–6 weeks (4–6 w) (Figure 4D).

**Fig. 5:**
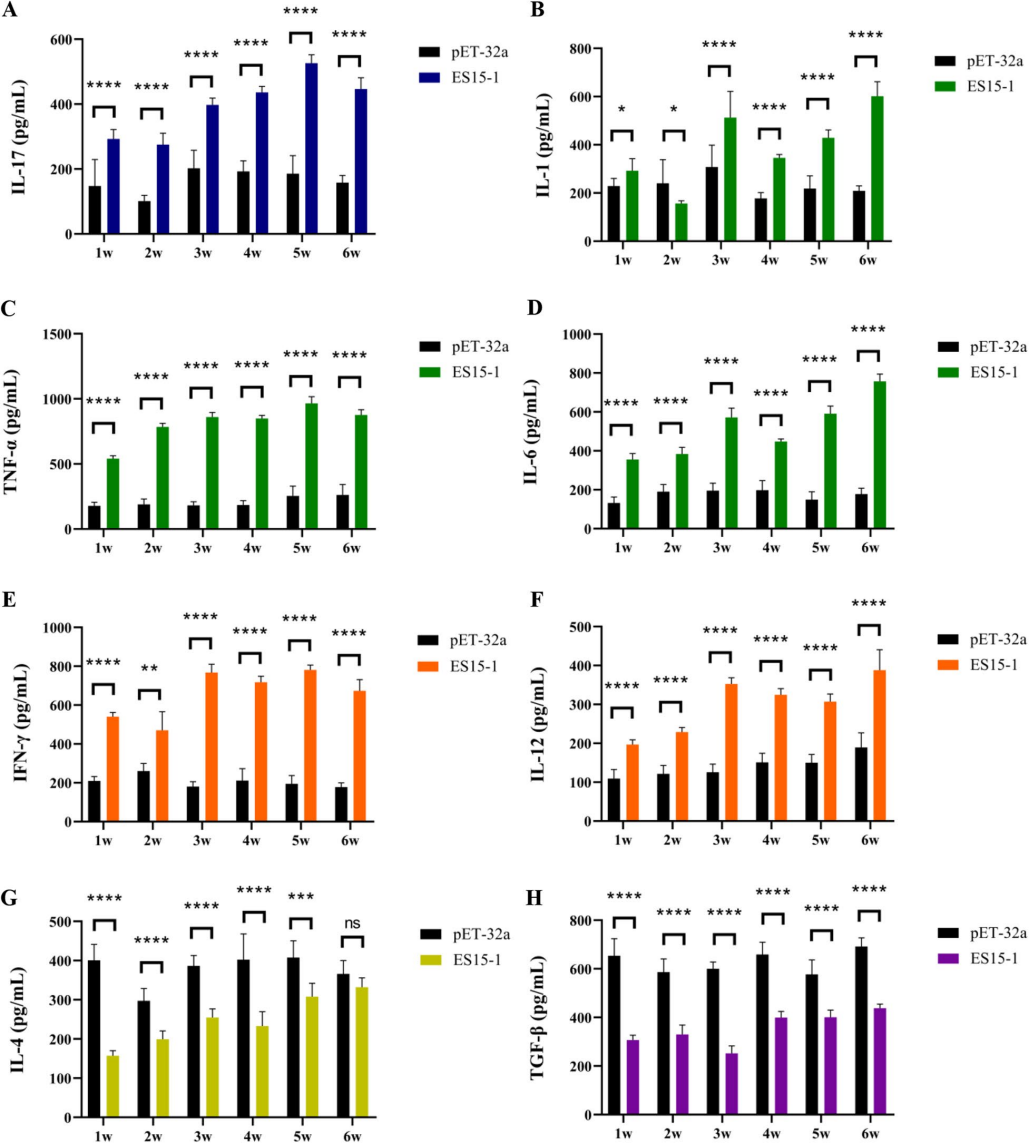
**ES15-1 regulated the secretion of Th17 related cytokines in mice.** IL-17 (**A**), IL-1(**B**), TNF-α (**C**), IL-6(**D**)**,** IFN-γ (**E**) and IL-12 (**F**) were upregulated, while IL4 (**G**) and TGF-β (**H**) were downregulated. Data are presented as Mean ± SEM. *P*-values < 0.05 (*), < 0.01 (**), < 0.001 (***), and < 0.0001 (****) were considered statistically significant. ns represented no significance.

ELISA analysis of serum cytokines in the group of PLGA-ES15-1 treated mice demonstrated that the level of inflammatory cytokine IL-17, TNF-α and IL-6 were consistently higher than those in control group across 1–6 w (Figure5A, C, D). The level of another inflammatory cytokine IL-1 significantly increased at 1 w and 3–6 w, but transiently reduced at 2 w (Figure 5B). In addition, the levels of IFN-γ and IL-12 in ES15-1 group were persistently upregulated throughout the 1–6 w period (Figure 5E, F). In contrast the level of IL-4 in serum was significantly lower than controls at 1–5 w, however, there is no significant difference observed at 6 w (Figure 5G). The level of TGF-β in serum persistently reduced across all weeks (Figure 5H).

### ES15-1 up-regulated IL-17 production in goat

**Fig. 6:**
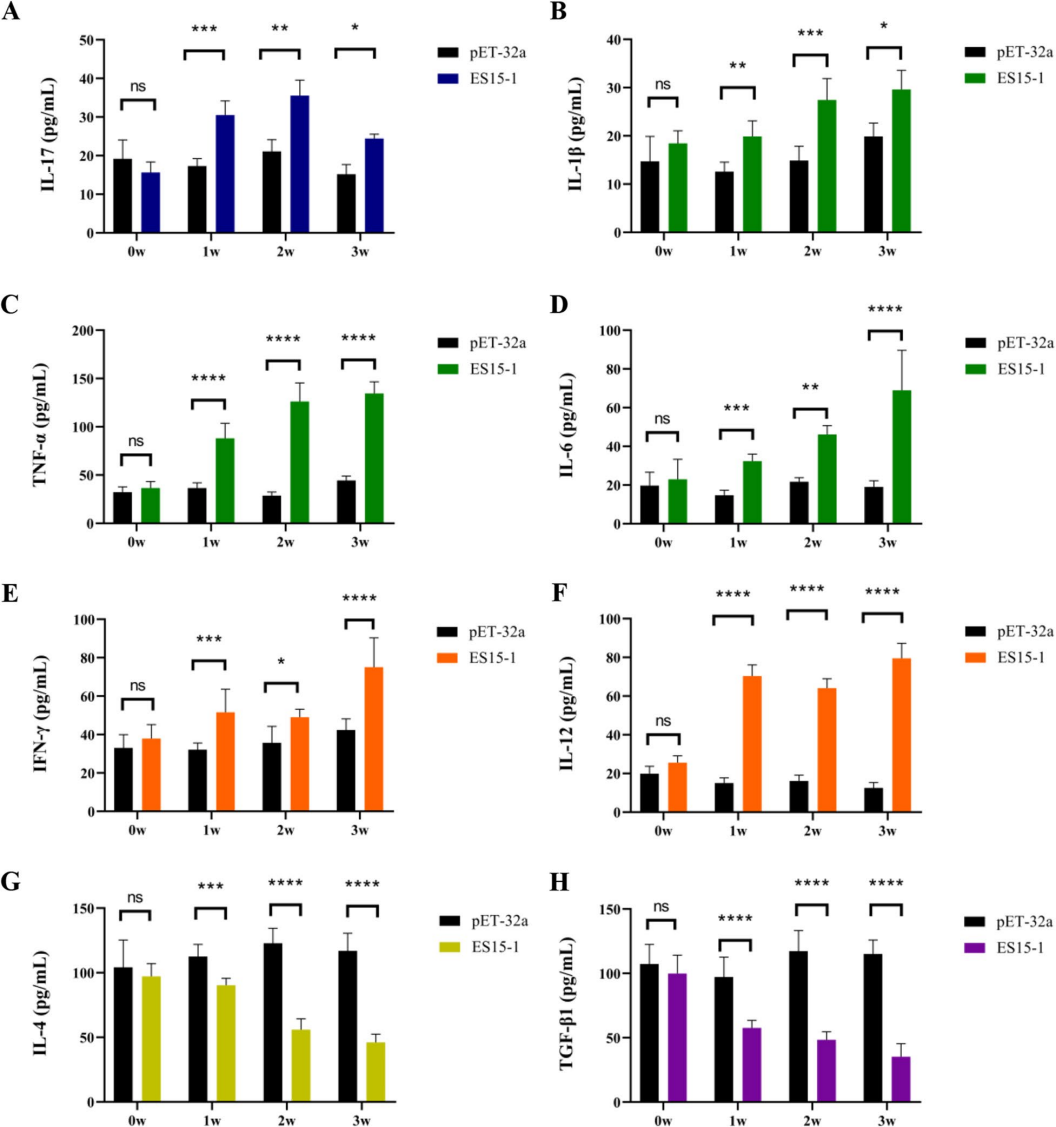
**ES15-1 regulated the secretion of Th17 related cytokines in goats**. IL-17 (**A**), IL-1(**B**), TNF-α (**C**), IL-6(**D**)**,** IFN-γ (**E**) and IL-12 (**F**) were upregulated, while IL4 (**G**) and TGF-β (**H**) were downregulated. Data are presented as Mean ± SEM. *P*-values < 0.05 (*), < 0.01 (**), < 0.001 (***), and  < 0.0001 (****) were considered statistically significant. ns represented no significance.

The results showed that there was no any significant difference in the serum cytokine levels between the ES15-1 immunized group and control group before immunization (0 w). IL-17 levels in the ES15-1 group were markedly higher than controls group from 1 to 3 w (Figure 6A). The level of inflammatory cytokines IL-1, IL-6 and TNF-α gradually significantly increased from 1 to 3w, compared to controls (Figure 6B–D). The secretion levels of Th1-polarizing cytokines IFN-γ and IL-12 were significantly increased at 1–3 w (Figure 6E, F). In contrast, the typical Th2 and Treg cytokines IL-4 and TGF-β showed sustained suppression from 1 to 3 w compared to controls (Figure 6G, H). These results suggest that PLGA-ES15-1induce transient but significant Th17 differentiation, peaking at 7 w. The elevated IL-17, TNF-α, and IL-6 levels align with robust Th17/Th1 activation. Meanwhile there is suppression of IL-4 (Th2) and TGF-β (Treg) suggest a shift toward pro-inflammatory immunity.

### ES15-1 induced partial protection in goat

**Fig. 7:**
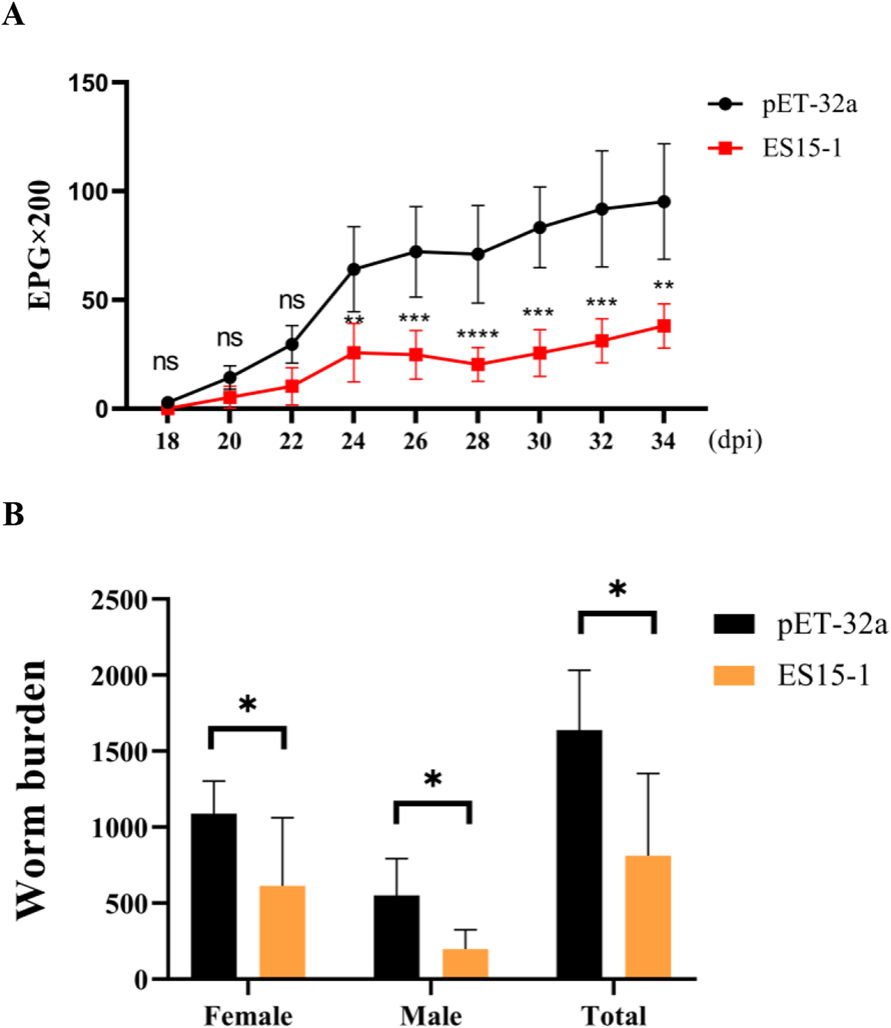
**Vaccination of ES15-1 result in the reduction of worm burden and egg shedding in goat**. **A** The dynamic of egg shedding in vaccination group (ES15-1) and control group (pET-32a). **B** Worm burden in the abomasum. Data are presented as Mean ± SEM. *P*-values < 0.05 was considered statistically significant. ns represented no significance.

FEC of the control group (pET-32a group) exhibited a progressive increase throughout the trial period. While the FEC of the ES15-1 group declined significantly during 45–49 d of the trial (24–28 dpi). *H. contortus* eggs were first detected in the feces of control group on day 39 post-trial initiation (18 dpi). In contrast, egg shedding in the ES15-1-vaccinated group exhibited delayed onset, with initial detection at day 41 (20 dpi). The ES15-1 vaccine significantly reduced FEC by 69.0% (*P* < 0.001) compared to the control group. (Figure 7A). Furthermore, ES15-1 immunization significantly protected against *H. contortus* challenge, demonstrating 43.65% (*P* = 0.047), 64.19% (*P* = 0.037), and 50.54% (*P* = 0.026) reductions in female, male, and total worm burdens, respectively, versus control animals (Figure 7B).

## Discussion

As specialized defenders against extracellular pathogens, Th17 cells constitute the early effector T cells involved in mucosal immune responses, particularly in barrier tissues such as the intestinal epithelium and respiratory mucosa exposed to a large number of potential pathogenic sites [29–31]. Th17 cells are potent inflammatory effector cells, that secrete inflammatory cytokines such as IL-17A, IL-17F, IL-6, IL-21, IL-22, IL-26, CX-CL8, TNF-α and GM-CSF, which plays important roles in the early stage of inflammation and inflammatory diseases [21, 32–34]. Previous studies have shown that IL-17 is significantly associated with experimental autoimmune encephalitis (EAE), asthma, rheumatoid arthritis (RA) and other autoimmune diseases [35–37]. IL-17 is an inflammatory cytokine produced primarily by activated T cells which can promote the activation of T cells, stimulate the production of multiple cytokines such as IL-6, IL-8, granulocyte–macrophage stimulating factor (GM-CSF), chemical activators and cellular adhesion molecule 1(CAM-1) by epithelial cells, endothelial cells and fibroblasts which leads to inflammation [38].

The differentiation of Th17 cells is regulated by transforming growth factor-beta (TGF-β), which induces the expression of the orphan nuclear receptor (RORγt). Additionally, IL-21 and IL-6, secreted by activated Th17 cells amplified this differentiation through autocrine signaling. Another pathway involves the activation of STAT3, which promotes Th17 lineage commitment. In this pathway, IL-6 and IL-1 synergistically drive T cells to express the IL-23 receptor [39]. The transcription factor IRF4 is a prerequisite for RORγt induction, as genetic ablation of IRF4 abolishes RORγt expression in naïve CD4⁺ T cells during Th17 differentiation [40–42]. Furthermore, the basic leucine zipper ATF-like transcription factor (BATF) is a master regulator essential for Th17 cell differentiation and transcriptional control of lineage-defining cytokines [43, 44]. The development of Th1 cells is driven by IL-12 and interferon-gamma (IFN-γ), with the transcription factor T-bet playing a critical role in regulating IFN-γ production [45, 46]. T-bet inhibits the transcription of RORγt by regulating the expression of IFN-γ, thereby suppressing Th17 cell differentiation [47]. Similarly, Th2 cells, whose differentiation is driven by IL-4, express the master transcription factor GATA3. GATA3 promotes IL-4 production while suppressing RORγt expression, thereby inhibiting Th17 cell differentiation [48–50]. Regulatory T cells (Tregs), characterized by the expression of the transcription factor Foxp3, also play a role in modulating Th17 responses [51, 52]. High levels of IL-6 and STAT3 signaling can induce hypoxia-inducible factor-1α (HIF-1α), which downregulates Foxp3 expression, thereby influencing the balance between Treg and Th17 cell populations [53–55].

Myeloid differentiation factor 88 (MyD88) is a critical adaptor protein in Toll-like receptor (TLR) signaling pathways. All TLRs, except TLR3, signal through the MyD88-dependent pathway [16, 56, 57]. Notably, IL-17 signaling shares similarities with TLR4 signaling, as both trigger activation of the nuclear factor-kappa B (NF-κB) and mitogen-activated protein kinase (MAPK) signaling pathways [17, 58]. Ligation of IL-17 to its cognate receptor (IL-17R) triggers recruitment of the adaptor ACT1 to the receptor’s SEFIR domain, facilitating TRAF6-dependent K63-linked polyubiquitination that initiates downstream NF-κB and MAPK signaling [59]. Phosphorylated TRAF6 dissociates from the receptor and interacts with transforming growth factor-β-activated kinase 1 (TAK1). Following cytoplasmic translocation, this complex nucleates higher-order assemblies with ubiquitin-binding enzymes to activate TAK1. The activated kinase then phosphorylates key elements in both MAPK and IKK complexes, initiating downstream signaling through NF-κB and MAPK cascades [18, 19]. The function of the peptide in the NF-κB/MAPK pathway was initially assessed by examining key signaling molecules (IL-1, MYD88, and NF-κB).

PLGA nanoparticles represent an ideal vaccine delivery platform due to their biocompatibility and controlled biodegradation with a diameter of 10~100 nm [60]. The ester bond of PLGA is hydrolyzed after contact with water to produce the degradation products lactic acid and glycolic acid monomers, which are metabolized to carbon dioxide and water through the tricarboxylic acid cycle [61]. When utilized as fixation devices or replacement implants in the musculoskeletal system (articular cartilage, meniscal tissue, and skeletal muscle), PLA-PGA copolymers exhibit satisfactory biocompatibility with no evident toxicity, despite the occasional occurrence of chronic inflammation marked by infiltration of macrophages, fibroblasts, multinucleated giant cells, and lymphocytes [62, 63]. Nanoparticles can encapsulate antigens including therapeutics agent, proteins, peptides, and plasmids to increase the accumulation of antigens in the body by enhancing permeability and retention effects [64, 65]. Therefore, PLGA is widely used for continuous, targeted and targeted drug delivery in clinical practice [66]. In our study, we aimed to induce higher level of IL-17 in mice and goat by coating ES15-1 with nanomaterials, given that Th17 cells are involved in the early stages of inflammation and inflammatory disease. Previous studies have shown that IL-17 plays an important role in host defense against *H. contortus* [62]. However, the mechanisms of Th17 cells in *H. contortus* infection remain unclear.

Through comprehensive bioinformatic analysis and empirical validation, we systematically evaluated the immunogenic potential of HcES15 by using epitope prediction, antigenic index calculation, and secondary structure analysis. Subsequent segment expression and incubation with PBMCs revealed distinct immune functions across different regions of HcES15. Specifically, peptides ES15-1 and ES15-3 significantly stimulated IL-17 transcription, whereas ES15-2 exhibited inhibitory effects. We selected ES15-1, a peptide predominantly upregulating IL-17, as the primary focus of our study. In addition to analyzing cytokines in the Th17 cell-related pathway in vitro, we also examined key transcription factors associated with Th1, Th2, and Treg differentiation pathways. Our findings demonstrated that STAT1 (Th1) transcription was suppressed, while Foxp3 (Treg) was upregulated at 6 h post-incubation of ES15-1 with PBMCs, further supporting the early involvement of Th17 cells in inflammatory responses. Flow cytometry analysis revealed that ES15-1 significantly induced Th17 cell differentiation in mouse spleen at 2w and 3w post-treatment. In both murine and caprine models, ES15-1 effectively stimulated the secretion of Th1-associated cytokines (IFN-γ and IL-12) and pro-inflammatory cytokines (IL-1, IL-6, and TNF-α), while suppressing the secretion of Th2 and Treg associated cytokines (IL-4 and TGF-β). In murine trials, IL-17 secretion gradually increased over 6 weeks following a single immunization, peaking at 5 weeks before slightly declining at 6 weeks, indicating a sustained Th17 response. These results suggest that PLGA-coated ES15-1 induces prolonged Th17 cell differentiation in mice through slow antigen release, promoting IL-17 secretion and subsequent inflammatory responses. However, in caprine trials, IL-17 secretion displayed a decreasing trend at 3 weeks post-secondary immunization. We hypothesize that elevated levels of IFN-γ and IL-12 may partially inhibit the unclear expression of STAT3 and RORγt, thereby suppressing IL-17 secretion at this timepoint. This observation aligns with the concurrent increase in IFN-γ and IL-12 levels, suggesting a potential regulatory mechanism underlying the dynamic Th17 response. Th1 helper cells primarily mediate immunity against intracellular bacteria and protozoa. IL-12 and IFN-γ are the cytokines required for the development and differentiation of the Th1 cells, among which IL-2 plays the driving and inducing function, and IFN-γ plays the executive role. The key transcription regulatory factors of Th1 cells are T-bet, STAT4, and STAT1. Excessive activation of Th1 response can lead to macrophage mediated autoimmune diseases such as leprosy, tuberculin overreaction, or type 1 diabetes. However, the anti-infective effects of Th1 response against the intestinal parasitism remain poorly understood. Therefore, although both IL-12 and IFN-γ were significantly upregulated in mice and goats by ELISA, their role in resisting *H. contortus* infection require further investigation.

Our murine experiments demonstrated that while ES15-1 treatment significantly inhibited IL-4 secretion, a progressive increase in IL-4 levels was observed throughout the 1–6w. It suggests the potential establishment of a synergistic Th2/Th17 immune response during later stages. However, Th2 cells typically play a dominant protective role in anti-helminthic immunity. This may represent one of the underlying mechanisms contributing to the anti-*H. contortus* efficacy of ES15-1. Although the precise mechanisms underlying the interplay between Th17 and Th2 cells during *H. contortus* infection remain incompletely characterized, current research demonstrates that Th17 cells can potentiate Th2-type immune responses via IL-17E (IL-25) secretion, thereby modulating both the magnitude and polarization of host immune responses. IL-17 potently activates stromal cells such as epithelial cells and fibroblasts, inducing them to secrete various chemokines (e.g., CXCL1 and CXCL2) and pro-inflammatory cytokines (including IL-6 and TNF-α). These cytokines not only participate in innate immune responses but may also establish a specific inflammatory microenvironment that indirectly promotes the chemotaxis, recruitment, and functional activation of Th2 cells, thereby forming a synergistic Th17-Th2 immune regulatory network. This multi-layered immunomodulatory mechanism likely represents a crucial host defense strategy against specific pathogen infections [67, 68].

Furthermore, IL-17 can induce CCL20 expression, which recruits Th2 cells to infection sites [69]. IL-21 derived from Th17 cells directly promotes Th2 cell proliferation and differentiation while simultaneously enhancing IgE production by B cells, thereby strengthening anti-parasitic humoral immunity [70]. IL-22 serves dual functions: it enhances mucosal barrier function to restrict parasite invasion and promotes Th2 cell activation by modulating the local microenvironment [71, 72]. Additionally, Th17 cells can indirectly influence Th2 cell differentiation by regulating the function of dendritic cells (DCs) and other antigen-presenting cells [73].

Our study demonstrates that peptide ES15-1 effectively induces Th17 immune responses in vivo and in vitro. This response correlates with significant reductions in fecal egg counts and worm burdens following *H. contortus* challenge, indicating that ES15-1 acts as a potent Th17 activator and confers promising immune protection. Collectively, these findings establish a mechanistic foundation for developing vaccines against *H. contortus*.

## Data Availability

No datasets were generated or analysed during the current study.
